# Auditory Survey of Endangered Eurasian Bittern Using Microphone Arrays and Robot Audition

**DOI:** 10.3389/frobt.2022.854572

**Published:** 2022-04-06

**Authors:** Shiho Matsubayashi, Kazuhiro Nakadai, Reiji Suzuki, Tatsuya Ura, Makoto Hasebe, Hiroshi G. Okuno

**Affiliations:** ^1^ Graduate School of Engineering Science, Osaka University, Toyonaka, Japan; ^2^ Department of Systems and Control Engineering, School of Engineering, Tokyo Institute of Technology, Tokyo, Japan; ^3^ Honda Research Institute of Japan, Wako, Japan; ^4^ Graduate School of Information Science, Nagoya University, Nagoya, Japan; ^5^ Wild Bird Society of Japan, Tokyo, Japan; ^6^ Sarobetsu Eco Network, Toyotomi, Japan; ^7^ Graduate School of Informatics, Kyoto University, Kyoto, Japan

**Keywords:** localiaztion, robot audition, microphone array, nocturnal birds, Eurasian bittern, advertisement calls, automated acoustic monitoring

## Abstract

Bioacoustics monitoring has become increasingly popular for studying the behavior and ecology of vocalizing birds. This study aims to verify the practical effectiveness of localization technology for auditory monitoring of endangered Eurasian bittern (Botaurus stellaris) which inhabits wetlands in remote areas with thick vegetation. Their crepuscular and highly secretive nature, except during the breeding season when they vocalize advertisement calls, make them difficult to monitor. Because of the increasing rates of habitat loss, surveying accurate numbers and their habitat needs are both important conservation tasks. We investigated the feasibility of localizing their booming calls, at a low frequency range between 100–200 Hz, using microphone arrays and robot audition HARK (Honda Research Institute, Audition for Robots with Kyoto University). We first simulated sound source localization of actual bittern calls for microphone arrays of radii 10 cm, 50 cm, 1 m, and 10 m, under different noise levels. Second, we monitored bitterns in an actual field environment using small microphone arrays (height = 12 cm; width = 8 cm), in the Sarobetsu Mire, Hokkaido Island, Japan. The simulation results showed that the spectral detectability was higher for larger microphone arrays, whereas the temporal detectability was higher for smaller microphone arrays. We identified that false detection in smaller microphone arrays, which was coincidentally generated in the calculation proximate to the transfer function for the opposite side. Despite technical limitations, we successfully localized booming calls of at least two males in a reverberant wetland, surrounded by thick vegetation and riparian trees. This study is the first case of localizing such rare birds using small-sized microphone arrays in the field, thereby presenting how this technology could contribute to auditory surveys of population numbers, behaviors, and microhabitat selection, all of which are difficult to investigate using other observation methods. This methodology is not only useful for the better understanding of bitterns, but it can also be extended to investigate other rare nocturnal birds with low-frequency vocalizations, without direct ringing or tagging. Our results also suggest a future necessity for a robust localization system to avoid reverberation and echoing in the field, resulting in the false detection of the target birds.

## Introduction

Birds are indicators of biodiversity. Historically, bird censuses relied on the ability of human observers to identify species of interest based on sight and sounds at the census site (Ralph et al., 1997). A shortcoming of direct observation is the variation of detectability, which can be influenced by potential observer bias (Sauer et al., 1994). Monitoring difficulties and outcome uncertainties are multiplied for rare species because of low detectability tied to low abundance. Effective monitoring of rare bird species, however, is critical to ensure their conservation. For some territorial species, reactions to playback of species-specific sounds are often used to overcome this constraint by triggering aggressive responses (Sutherland et al., 2004). However, this technique using playbacks only works for certain species, while often silencing cryptic species. Paradoxically, these species are usually the target of conservation, and accurate measurement of demography, such as abundance, dispersal, and recruitment, are important for management plans. Such demographic information has been collected by catching, marking, and more recently by tagging logging devices, which involve a certain range of physical interferences. These techniques may be inappropriate for monitoring rare endangered species or species that are sensitive to human interference. Auditory monitoring has the specific merit of monitoring targets without human interference.

Of these rare and sensitive species, birds that have low-frequency vocalizations are particularly challenging to monitor. Their low-frequency sounds are extremely difficult to locate for human observers in the field. For example, sounds are scattered, attenuated, and reverberated by the topography and forest vegetation. In open wetlands, sounds can be reflected from the surface of water. In addition to these abiotic factors, many of their specific vocalizations occur only over a short period of time, like during the breeding period. Monitoring in remote fields at night or in crepuscular conditions is also a high-risk task for human observers.

Recent advances in bioacoustics and microphone arrays, provide a powerful tool for the auditory monitoring of birds. The potential merit of microphone arrays lies in detecting the position of sound occurrence, or the direction of arrival (DOA) of sound sources. Advances in acoustic processing, particularly in microphone arrays, have provided alternative methods to passively monitor bird behaviors (Blumstein et al., 2011; Mennill 2011). Microphone arrays, or a set of single microphone arrays have been used to acoustically monitor the territory of Mexican-ant-thrush (*Formicarius moniliger*) (Kirschel et al., 2011), the duetting behavior of rufous-and-white wrens (*Thryophilus rufalbus*) (Mennill and Vehrencamp 2008), the position of warblers in flight (Gayk and Mennill 2019), and the population density of ovenbirds (Dawson and Efford 2009). However, despite their potential advantages, microphone arrays are not widely adopted in the field of bird monitoring because of the limited availability of the required software and hardware.

To overcome these challenges, we developed HARKBird, a portable recording system that monitors and analyzes birdsongs. HARKBird consists of a standard laptop computer, an open-source robot audition system called HARK (Honda Research Institute, Audition for Robots with Kyoto University) (Nakadai et al., 2010), and commercially available low-cost microphone arrays. The software for HARKBird is entirely composed of a series of Python scripts with modules (e.g., wxPython and PySide) and other standard sound processing software (e.g., SOX, arecord, and aplay). All these software packages can operate in the Ubuntu 12.04 Linux operating system, where the latest HARK and HARK-Python scripts have been installed. The algorithm for sound source localization is based on the MUSIC method (Schmidt, 1986) and multiple spectrograms with a short-time Fourier transformation (Nakamura et al., 2013). Localized sounds are separated into multiple songs using the geometric high-order decorrelation-based sound separation method in real time (Nakajima et al., 2010). See Nakadai et al. (2010) and Suzuki et al. (2017) for HARK and HARKBird, respectively. Previously, we monitored great reed warblers *(Acrocephalus arundinaceus*) (Matsubayashi et al., 2017), bush warblers (*Horornis diphone*) (Suzuki et al., 2018), and nocturnal species such as owls (*Strix Uralensis*) and crepuscular ruddy-breasted crake (*Porzana fusca*) (Matsubayashi et al., 2021) using microphone arrays and HARKBird. A question remained regarding the frequency range defined by the upper and lower limiting frequencies, which were in the range of 500 and 3,000 Hz in the above examples.

Generally, the limiting frequencies of microphone arrays are linked to their size, compared with the wavelength of sound. Because the wavelength is inversely proportional to the frequency, it becomes progressively longer at lower frequencies. In other words, a small-sized microphone array is better suited for localizing higher-frequency sounds, whereas large-sized microphone arrays are better suited for localizing low-frequency sounds. The sensitivity of the microphone is also related to its size, which also affects its dynamic range.

**FIGURE 1:**
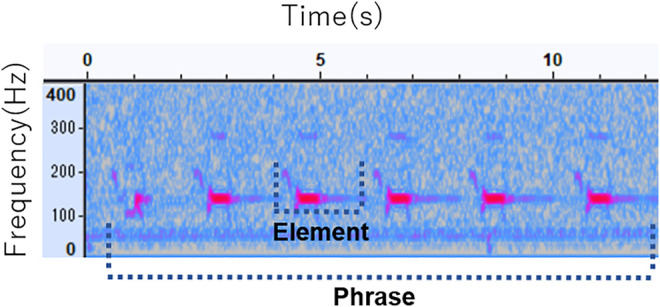
Structure of Eurasian Bittern’s call.

Here, we report on the feasibility of using relatively small-sized, 8-channel microphone arrays and HARKBird to acoustically monitor nocturnal birds with extremely low frequencies. Our recording target was the Eurasian bittern (*Botaurus stellaris*). It is a rare wetland bird, nationally protected as an endangered species in Japan; thus, accurate monitoring, for example, counting accurate numbers and social interactions are both important for decisions concerning the habitat management of this species. Eurasian bitterns are highly cryptic and difficult to observe. Males characteristically loud, monotonous, emit advertisement vocalizations called booming, at low frequency. An example of their boom, mostly in the frequency range of 100–150 Hz, is shown in Figure 1. Because of their secretive nature and inhabiting remote wetlands covered with thick vegetation, their booming calls are often the only sign of the presence of these birds for a human observer.

Previously, McGregor and Byle (1992) surveyed bitterns using stereo microphones in 1975–1976, and 1988–1989, concluding that the booming calls of bitterns are individually distinctive based on time and frequency. However, it was mentioned that the variability of the prelude, or so-called bad poor booms, can decrease the detection rates. More recently, Adamo et al. (2004) examined intra- and inter-specific booming of 18 radio-tagged bitterns using stereo recordings and found that vocalizations of the same male were not stable over time. Frommolt and Tauchert (2014) identified individual bitterns based only on spectrogram template matching derived from four four-channel microphone arrays, each of which were 30 cm apart, to monitor the population size. Wahlberg et al. (2003) also estimated the location of the bittern using four microphone arrays, each of which was placed 65 and 294 m apart, with a spatial localization accuracy of approximately 100 m.

The aim of this study was two-fold. First, we assessed the feasibility of portable microphone arrays to acoustically monitor low-frequency vocalizations by simulating the size of the microphone array under different noise levels. In this analysis, we examined both the temporal and spectral resolutions of localization. Second, we aimed to monitor bitterns in an actual field environment using relatively small microphone arrays.

## Methods

### Simulation

**FIGURE 2:**
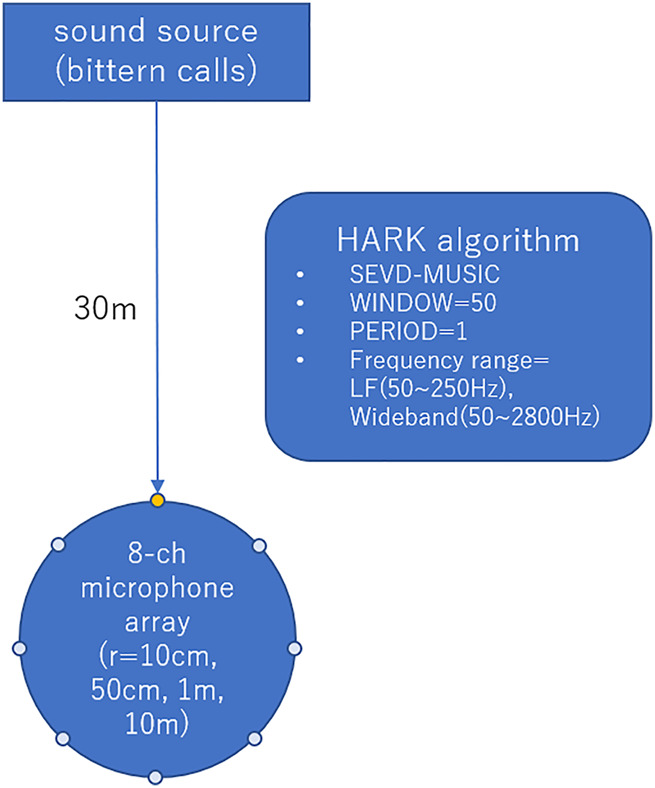
Schematic illustration of numerical simulation.

We examined the sound source localization by numerical simulation. Figure 2 shows a schematic illustration of this simulation. For this simulation, we assumed an 8-ch circular microphone array in a free acoustic space, with radius of 10 cm, 50 cm, 1 m, or 10 m. On all microphone arrays, 8 microphones were placed on the periphery at the angular interval of 45°. The sound source we used for simulation was a 10-s recording of the actual bittern booming. Owing to the low intensity of the original sound source, we increased its sound pressure by 12 dB using the SOX (version 14.4.2.), sound processing software (http://sox.sourceforge.net/). The sound source was assumed to be located 30 m away from the center of the microphone array in the horizontal direction at 0° with respect to the radial microphone array coordinates. The input to the microphone array was simulated by convolution, according to the geometrical relationship between the sound source and the microphone array. The distance between the microphone array and the sound source was set to 30m, for the clear recording of the sound. The distance is also practical, as we can position the recording device without interfering the target activity in the field. In this simulation, we additionally considered ambient noise. We added a diffused white noise to the convoluted sound source so that the signal-to-noise ratio (SNR) can attain 20, 10, and 0 dB. For analog-to-digital conversion, we used 16 bit and 16 kHz sampling.

We calculated the MUSIC spectrum values for two frequency ranges: the lower one specifically targeting bitterns ranging between 50–250 Hz (LF), and the other for wider bandwidth, ranging between 50 and 2,800 Hz (WB). We then used the MUSIC method to estimate the DOA by calculating the eigenvalue decomposition of the correlation matrix among the input signal channels. The MUSIC method uses the transfer function between a sound source and an M-channel microphone array, as a prior information, defined as,

$$H\left(\theta ,\omega \right)={\left[h_{1}\left(\theta ,\omega \right),\cdots ,h_{i}\left(\theta ,\omega \right),\cdots ,h_{M}\left(\theta ,\omega \right)\right]}^{T}$$ (1)

where $h_{i}\left(\theta ,\omega \right)$ denotes the transfer function between the sound source, S(θ) , from the direction, θ , in the microphone array polar coordinates and the i-th microphone. *ω* indicates frequency.

The M-channel input signal vector in the frequency domain is denoted as,

$$X\left(\omega ,f\right)={\left[X_{1}\left(\omega ,f\right),\cdots ,X_{i}\left(\omega ,f\right),\cdots ,X_{M}\left(\omega ,f\right)\right]}^{T}$$ (2)

where $X_{i}\left(\omega ,f\right)$ is a spectral component of the i-th microphone at the f-th time frame and at frequency ω , which can be obtained by performing short-term Fourier transform (STFT) to the input acoustic signal recorded with the i-th microphone of the microphone array.

The correlation matrix is calculated from X(ω,f) by,

$$R\left(\omega ,f\right)=\frac{1}{W}\overset{W}{\underset{i=0}{\sum }}X\left(\omega ,f+i\right)X^{*}\left(\omega ,f+i\right)$$ (3)

where ()*∗* represents the conjugate transpose operator; and W represents the window length for time averaging.

The eigenvalue decomposition is then performed for the correlation matrix R(ω,f) as,

$$R\left(\omega ,f\right)=E\left(\omega ,f\right)\Lambda \left(\omega ,f\right)E^{-1}\left(\omega ,f\right)$$ (4)

where $E\left(\omega ,f\right)=\left[e_{1}\left(\omega ,f\right),e_{2}\left(\omega ,f\right),\cdots ,e_{M}\left(\omega ,f\right)\right]$ represents the eigenvalue matrix comprising the Eigen vectors perpendicularly intersecting each other; and Λ(ω,f) is the diagonal matrix comprising the eigenvalues in descending order.

Larger eigenvalues indicate higher power sound sources, that is, the target signal; whereas the lower eigenvalues correspond to the noise signals. By using the threshold, $N_{s}$ , indicating the number of sound sources, the *M-dimensional* space of the correlation matrix is decomposed into the signal subspace and noise subspace. As performing the eigenvalue decomposition through every frame is computationally expensive, it was calculated only once in several frames in our implementation with the parameter called PERIOD.

The MUSIC spatial spectrum was calculated by,

$$P\left(\theta ,\omega ,f\right)=\frac{H^{*}\left(\theta ,\omega \right)H\left(\theta ,\omega \right)}{\sum_{i=N_{s}+1}^{M}\left|H\left(\theta ,\omega \right)e_{i}\left(\omega ,f\right)\right|}$$ (5)

As the eigenvectors from $e_{N_{s}+1}\left(\omega ,f\right)$ to $e_{M}\left(\omega ,f\right)$ belong in the denominator, that is, the noise subspace, the inner product of H(θ,ω) and these eigenvalues will be 0 when θ directs to the sound direction. P(θ,ω,f) then becomes infinity. Therefore, P(θ,ω,f) and the power of the sound source exhibit a non-linear correlation. Finally, sound source localization was achieved by identifying the peaks on the MUSIC spatial spectrum.

We used HARK 3.1.0 as the implementation for sound source localization and HARKTookl5-GUI 3.1.0 for the calculation of the transfer function between the sound source and the microphone array. We used an 8-channel microphone array (M=8). For STFT explained in Eq. 2, a window size of 512 samples (32 ms) and a window shift of 160 samples (10 ms) were used. This indicates that each frame of the input sound is represented as a matrix comprising 8 channels of 257 dimensional vectors. The number of channels corresponds to that of the microphones, whereas the number of dimensions corresponds to that of the frequency bins ranging between 0 and 8,000 Hz, which indicates that the bandwidth of each frequency bin is 31.25 Hz (8,000/256). As the frequency range for sound source localization was limited to 50–250 Hz, seven frequency bins from the 3^rd^ (62.5 Hz) to the 9^th^ (250 Hz) were used in this simulation.

As the first step of the MUSIC algorithm, the correlation matrix was calculated for each frequency bin in each frame, which produced an 8 × 8 correlation matrix for each frequency bin. Subsequently, to increase the stability of the correlation matrix, temporal integration was performed by averaging 50 frames of correlation matrices (W = 50). As the interval between two consecutive frames was 10 ms according to the window shift of STFT, the correlation matrix for one frame input was calculated from the 500 ms input signal. The number of sound sources, $N_{s}$ , to calculate the MUSIC spatial spectrum was set to 1.

### Study Site and Bird Recording

The study site was located in the Sarobetsu Mire, in the northernmost area of Hokkaido Island, Japan (Figure 32). The Sarobetsu mire encloses 6,700 ha of freshwater lakes and wetlands, some of which belong to the Rishiri-Rebun-Sarobetsu National Park and partially protected under the Ramsar convention since 2005 (Saito et al., 2008). The area is a known breeding ground for endangered birds, such as the Eastern marsh harrier (*Circus spilonotus*), Japanese red-crowned crane (*Grus japonensis*), and Yellow-breasted bunting (*Emberiza aureola*).

**FIGURE 3:**
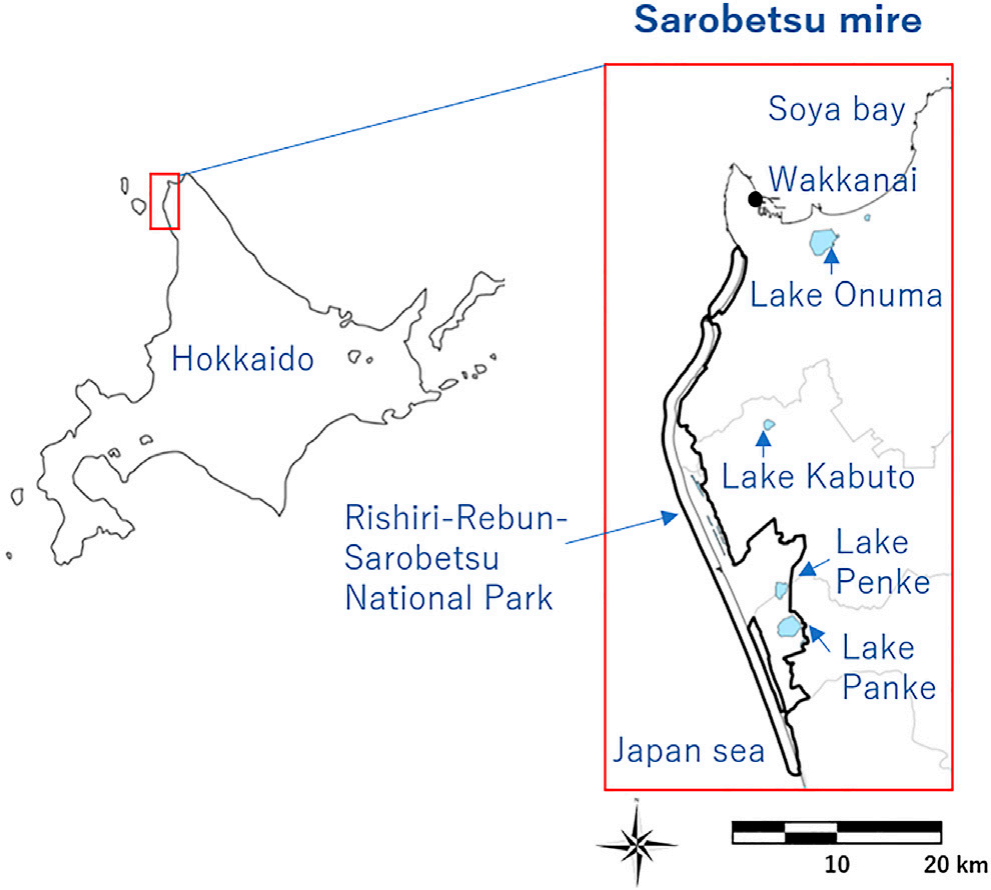
Location of the study area.

**FIGURE 4:**
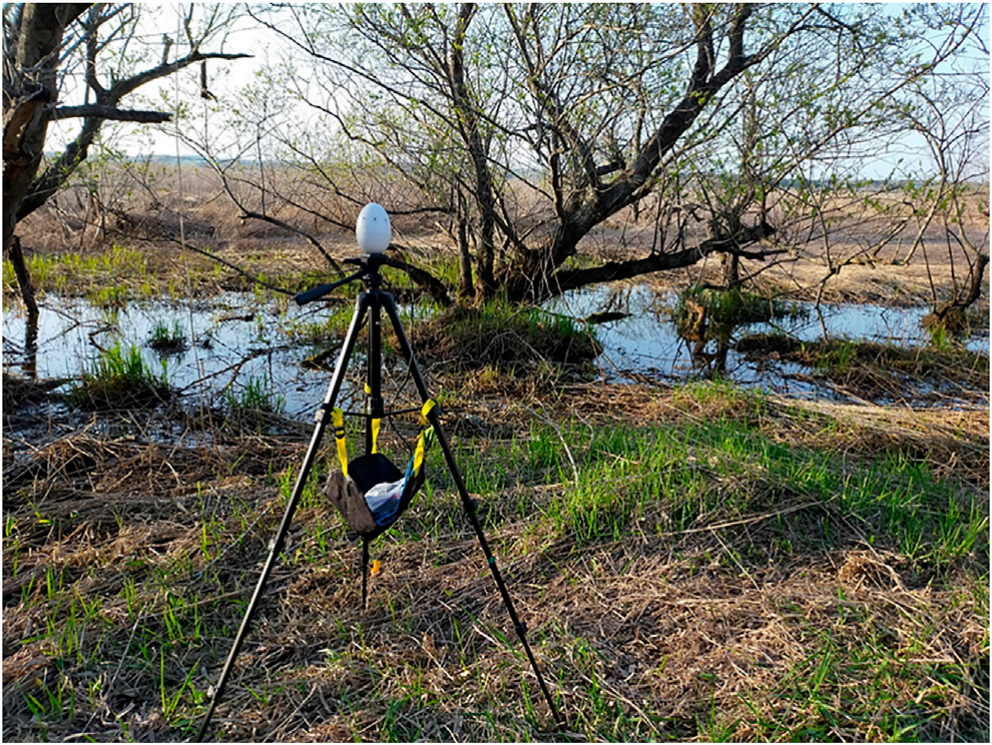
Photograph of the 8-channel microphone array.

We continuously recorded bitterns from 17:00 to 10:00 on 13 May 2021 during the peak breeding season. Bitterns were recorded using 8-channel microphone arrays (System in Frontier, TAMAGO-03, Japan). Eight microphone nodes were arranged horizontally at every 45° angle along the periphery of the egg-shaped body (height = 12 cm; width = 8 cm). This structure allowed us to estimate the azimuthal DOAs of the sound sources. The data was acquired for each channel at 16,000 Hz and at a resolution of 24 bits. We placed the microphone array on a tripod approximately 1.5 m above the ground, and connected it to a small PC (Raspberry-pi 4, United Kingdom) and a mobile battery using USB cables (Figure 4). We synchronized all the clock of all PCs by tethering them to a cell phone. A total of five microphone arrays, were placed at the interval of approximately 300–500 m, along the edge of the wetland.

The wetland is a freshwater back marsh of the Sarobetsu river, with the maximum depth of 2 m. The perimeter of the water surface is approximately 3.5 km (Saito et al., 2008). The wetland contains s*phagnum spp.* communities, surrounded by reed species and Asian skunk cabbage (*Lysichiton camtschatcensis*), under the canopies of alder (*Alnus japonica*).

The night had almost no wind. To sample vocalizations during the hours of darkness, while avoiding active hours of the grizzly bears (*Ursus arctos*), recording experiments in the study area were conducted mostly unattended by human observers. Human observers attended only for the first few minutes to check whether the equipment started correctly.

We silently monitored the birds non-invasively, apart from potential nesting sites and without playback experiments using loudspeakers to trigger territorial responses. This study was conducted with the approval of the Hokkaido Regional Environment Office and Osaka University’s Animal Experiment Ethics Review Committee.

### Localization

We estimated the DOA of the sound source acquired offline from the microphone array using HARKBird. Details of the major localization parameters are summarized in Table 1. We adjusted each parameter value by visually examining the spectrogram of each localized sound. We also auditorily inspected each of the localized sounds and manually removed sounds that were not bittern calls.

**TABLE 1:** Localization parameters of HARKBird.

Parameter	Unit	Value	Explanation
PAUSE_LENGTH	Millisecond	500	Previous section length of sound source
MIN_SRC_INTERVAL	Degree	10	A threshold for angle difference to be regarded as the same sound source
PERIOD	Number of frames	16	Cycle to calculate localization results
THRESH		13	A threshold to separate the sound source and noise
UPPER BOUND FREQUENCY	Hz	200	Upper bound frequency
LOWER BOUND FREQUENCY	Hz	100	Lower bound frequency
NUM_SOURCE		1	The number of sound sources

## Results

### Simulated Temporal and Directional Resolutions

**FIGURE 5:**
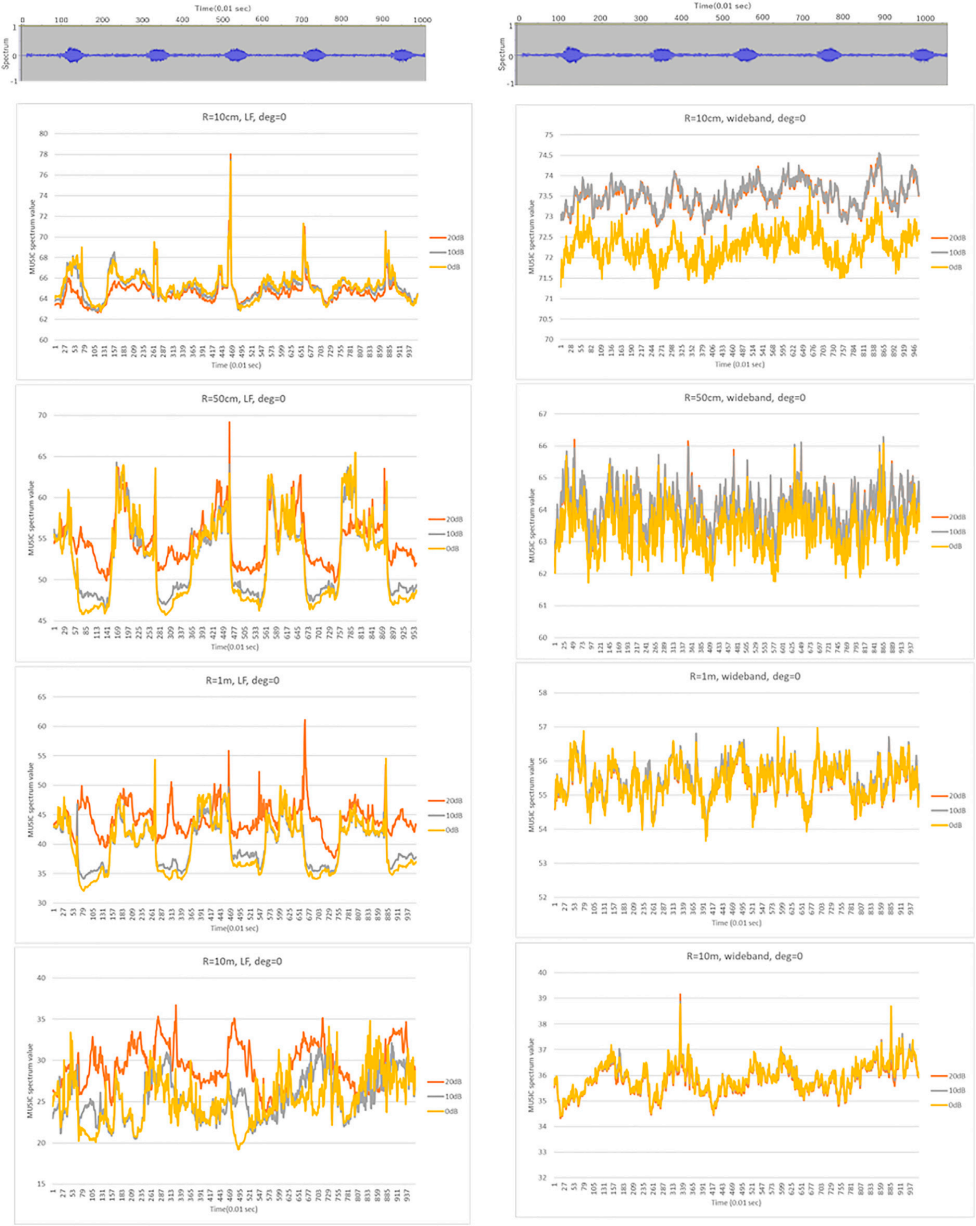
MUSIC spectrum in time for LB (left) and WB (right). The top subgraphs show the input signals in time domain, and the remaining subgraphs illustrate the MUSIC spectrum for the target direction (0°) over all frames for the corresponding frequency range. The horizontal and vertical axes are the simulation frame and MUSIC spatial spectrum values. The peaks in each subgraph show the presence of the sound source.

**FIGURE 6:**
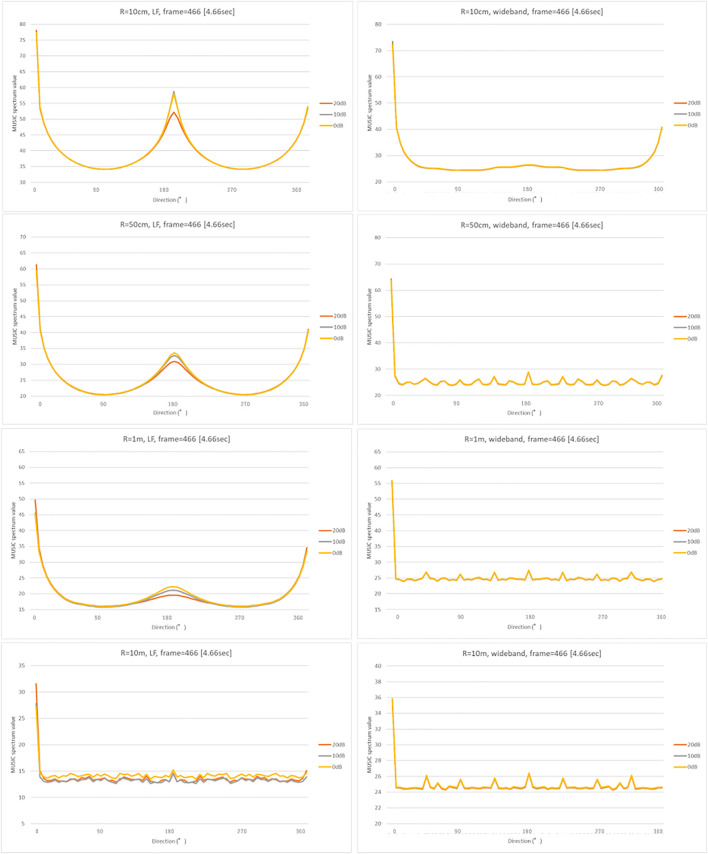
MUSIC spatial spectrum for LB (left) and WB (right). The vertical and horizontal axes indicate the direction and MUSIC spatial spectrum value, respectively. The conditions of the 1^st^-4^th^ subgraphs correspond to those of the 2^nd^-5^th^ subgraphs in Figure 5.

Figures 5, 6 show the results of the numerical simulation for LF (left) and WB (right), respectively. The top subgraphs in Figure 5 show the input signals in the time domain, and the remaining subgraphs illustrate the MUSIC spectrum for microphone arrays of radii 10 cm, 50 cm, 1 m, and 10 m. The colored line graphs in each subgraph show the results under three different noise levels (20, 10, and 0 dB). Figure 6 shows the MUSIC spatial spectrum at the 466-th time frame, which obtained the highest peaks as shown in Figure 5. The conditions of the 1^st^-4^th^ subgraphs if Figure 6 correspond to those of the 2^nd^-5^th^ subgraphs in Figure 5.

As expected, narrowing to the frequency range of the target sound, that is the low frequency call of bittern, increased the temporal detectability. For example, the peaks on the WB in Figure 5 were generated irrelevant to the presence of the actual bittern calls in the given timeframe, shown on top. In contrast, the timing of certain peaks in LF matched the actual bittern calls, indicating higher temporal detectability. Surprisingly, the smallest sized microphone array exhibited the highest temporal detectability, indicated by a higher match between the peaks on the graph, corresponding to the timing of actual bittern calls. The detectability decreased with increasing size of microphone array, that is, the deviation between the simulated peaks and the timing of booming became increasingly larger. The peaks were marginal for the microphone arrays of radius = 10 m. The influence of noise on the detectability were unclear. The detectability was higher under a quieter environment (with low noise level) for the smallest sized microphone array (radius = 10 cm), whereas this trend became unclear with low detectability associated with larger microphone arrays.

In contrast, the directional detectability increased with the size of microphone arrays (Figure 6). There was a ghost peak at 180°, or opposite to the target sound source direction (Figure 2), on the small-sized microphone arrays. Presumably, this ghost peak was coincidentally generated by the proximity to the transfer function for the opposite side, although this phenomenon requires to be investigated in greater detail in future studies. The size of the ghost peak was remediated by increasing the size of the microphone arrays. The results additionally showed that the slope became sharper for large-sized microphone arrays at approximately 0°, which corresponds to the main lobe in beamforming. Despite these ghost peaks generated coincidentally, the width of the main lobe was much narrower on the large-sized microphone arrays, indicating a higher spatial resolution. The detectability was high under low noise levels as expected. However, the performance was approximately maintained up to 0 dB, which is an established advantage in MUSIC algorithms.

### Detection of Booming Calls in the Field

**FIGURE 7:**
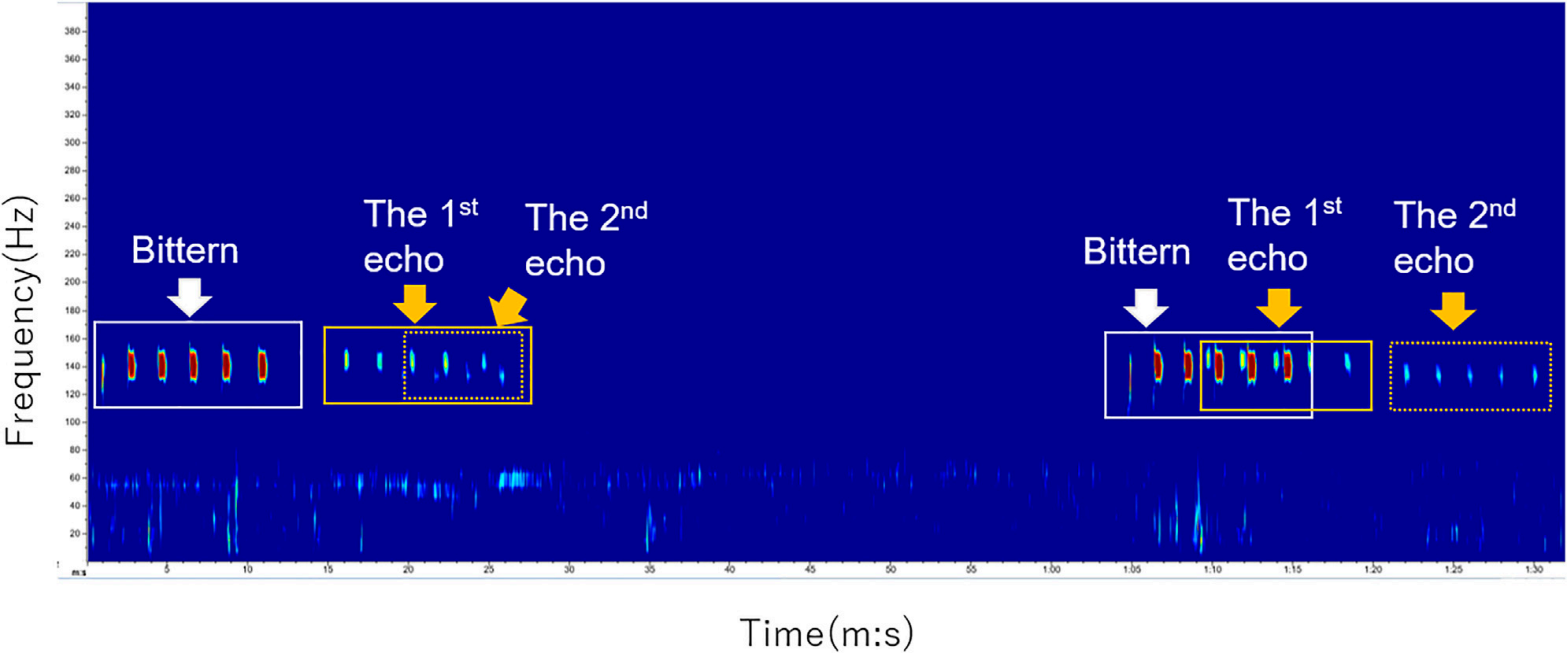
Example spectrograms of bittern calls and echoes.

The relative position of the microphone arrays to the target birds, the surrounding vegetation, and the water surface resulted in reverberation and echoes of actual bittern calls. Figure 7 shows spectrograms of bittern-like sound derived from an 8-channel microphone array, which was set on the edge of the water body adjacent to the forest. Two phrases, the one starting at 0:01 and the other starting at 1:05, both of which consisting of six syllables, were actual bittern songs, while others were echoes of those originals. The original calls were echoed twice each time. The 1st echo kept the same number of syllables, that is, six syllables in this case, while the number of syllables decreased in the 2^nd^ echo, thus fragmented. The number of syllables is kept in the 1^st^ echo, probably because the original calls vocalized in the wetland were first reflected directly by the thick trees and bushes behind the microphone array. Differences in the time delay between these two sets of echoes were presumably caused by the face orientation of the long-neck bittern and environmental reverberation, for example, trees, vegetation, and the extent of water surface, all of which could generate a complex reflection effect. The reflection effect also caused differences in the degree of sound fragmentation in the 2^nd^ echoes.

**FIGURE 8:**
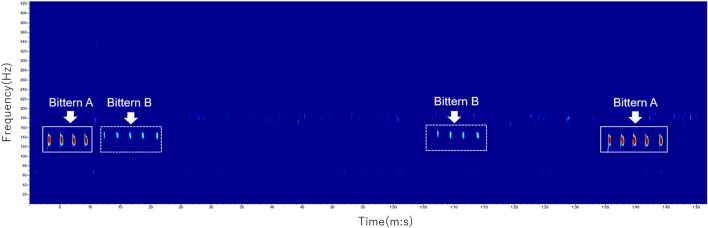
Example spectrograms of the two bitterns’ calls.

The maximum detection distance in open area was approximately 250 m. Although the sound attenuation caused by vegetation was minimum in open area, we used recordings from a site unaffected by echoes for further analysis, because distinguishing the echoes of the original and actual bitterns far away was challenging when random time delays occurred. Echoes were less severe in a bush along a wetland covered with thick vegetation. Figure 8 shows an auditory scene when two bitterns were booming alternately. In this two-minute recording, Bitten A first started a booming phrase consisting of four elements near the microphone array. Four seconds later, Bittern B at some distance from the microphone array vocalized a booming phrase consisting of five elements. Bittern B then boomed again approximately 40 s later, a phase consisting of four elements. After 20 s, Bittern A boomed again, a phase consisting of five elements. Note that the previous boom with five elements occurred approximately six min before the recording of Bittern B at 0:12, so that they were less likely to be echoes. Similarly, one minute of silence between Bittern A starting at 0:03 and Bittern B starting at 01:07, seemed too long to make the subsequent one an echo, although both consisted of four elements. Additionally, the results matched field observations we made at the beginning of the recording which was two hours before this scene.

### Localization of Bitterns

**FIGURE 9:**
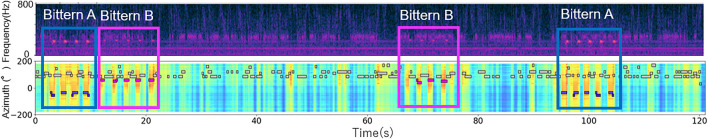
Examples of localized sound including the two bitterns’ calls.

We detected at least two booming males based on the differences in the DOAs derived from each microphone array. The localization parameters are listed in Table 1. Figure 9 shows examples of localized calls of the two bitterns, as described in Figure 8. At 18:30 on May 13th, Bittern A and B were localized at 45° and 350°, respectively, with respect to the microphone array.

We detected 528 calls of Bittern A and 260 calls of Bittern B during the observation period at 4:45, as described above. The temporal localization accuracy between 17:30 and 20:30 when vocalization was most active was summarized in Table 2. The comparison between the localized and annotated call duration showed that there was a difference in the localization accuracy. It was highly influenced by the surrounding noise and the distance between the microphone array and target individual. The localization accuracy was higher for Bittern A, which was singing in closer proximity to the microphone array between the range of 64.4–95.2% during the peak hours. High localization accuracy indicates the high reliability of the automated monitoring data for further analyses. In contrast, the localization accuracy was low for Bittern B, which was booming farther away. Even after other songbirds stopped singing at around 18:00, the localization accuracy remained low, at 27.2% between 18:30 and 19:30, and 46.5% between 19:30 and 20:30. Additionally, false detection was caused by environmental noises such as rustling of trees and waders singing in a direction similar to that of the bittern at the beginning of the evening.

**TABLE 2:** Temporal localization accuracies for each bittern. Localization accuracy was calculated by dividing the actual duration by the localized duration. The actual duration was inspected manually.

	Bittern A			Bittern B		
Time	17:30–18:30	18:30–19:30	19:30–20:30	17:30–18:30	18:30–19:30	19:30–20:30
Localized duration(s)	61.6	109.3	147.0	385.6	151.8	256.2
Actual duration(s)	39.7	104.1	126.4	22.4	41.3	119.0
Localization accuracy (%)	64.4	95.2	86.0	5.8	27.2	46.5

**FIGURE 10:**
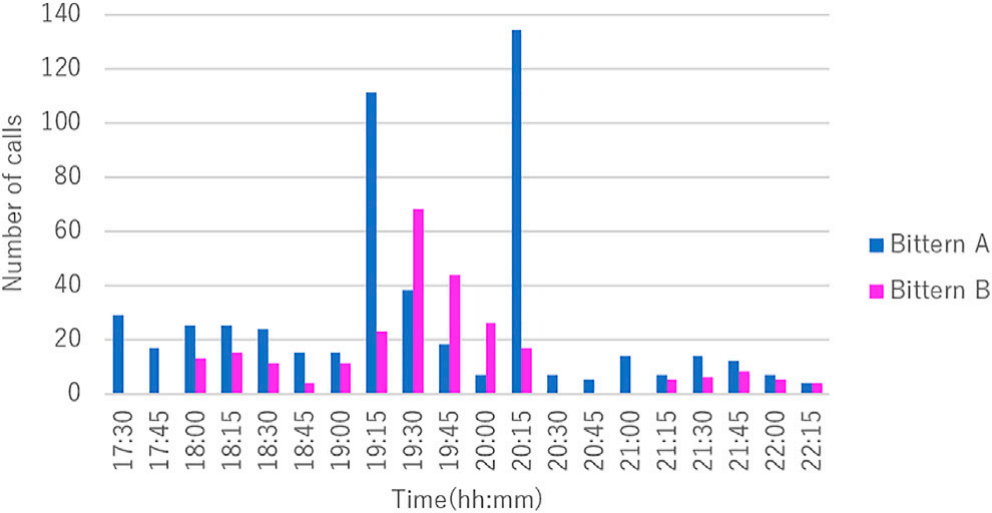
Temporal distribution of the two bitterns’ calls.

Figure 10 shows the temporal distribution of the localized calls of the two bitterns at the same time frame. Bittern A boomed throughout the sampling period. The number of calls peaked twice, at 19:15 and 20:15, which were 21.0 and 25.4% of the total calls of Bittern A, respectively. On the other hand, Bittern B started booming at 18:15 with increasing frequency, with its peak at 19:45, being 26.15% of the total of Bittern B. Bittern B became silent approximately 60 min starting at 20:00. The number of calls was lower after silence. Both bittern’s calls were most active around sunset at 19:53, constituting 63.45% of the total call of Bittern A, and 72.69% of that of Bittern B occurred within 60 min before and after sunset at 19:53. Notably, the peak timing of Bittern B’s call that occurred between 19:45 and 20:00 matched the timing of sunset. The vocalization frequency dropped after this peak time for both bitterns.

**FIGURE 11:**
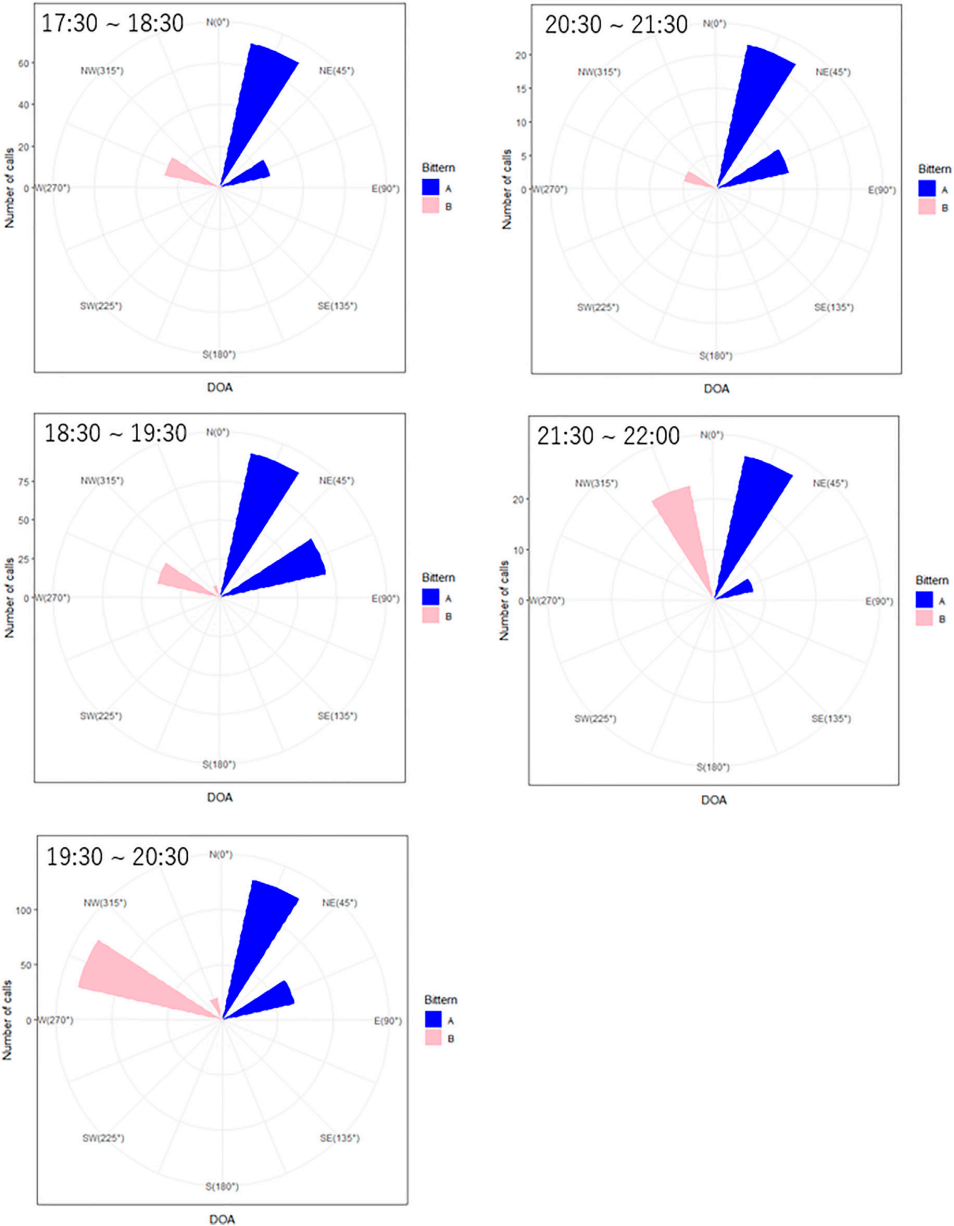
Circular histogram of the direction of arrival (DOA) of the two bitterns’ calls.

**FIGURE 12:**
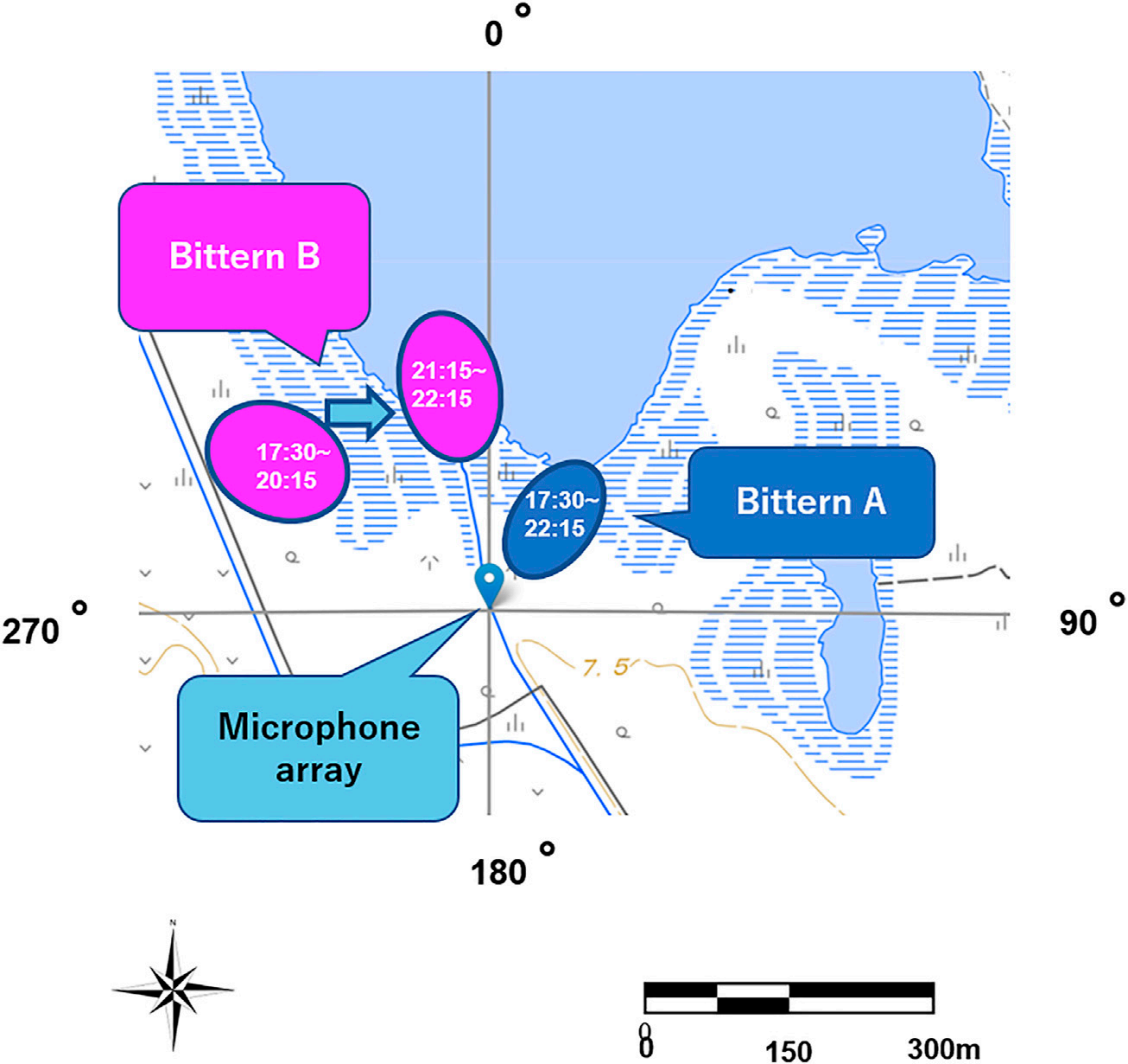
Estimated location of the two bitterns relative to the microphone array.

The numbers and DOAs corresponding to each bittern boom are shown on a circular histogram, during the same time intervals are summarized in Figure 11. The location of the microphone array and the approximate location of the two bitterns based on the DOAs and the amplitude of their calls are shown on a map in Figure 12. Bittern A was relatively stationary, vocalizing at approximately 45° or northeast of the microphone array. On the other hand, Bittern B, which was booming at 56.25°, or northwest of the same microphone array between 17:30 and 21:15, including 45 min of complete silence, and drastically changed its location at 21:30, to 11°, or approximately north of the microphone array.

## Discussion

The simulation results revealed two technical limitations of the recording device used in the field. First, ghost peaks were generated on the opposite side (180°) of the sound source on smaller microphone arrays, although it had a higher sensitivity for detecting MUSIC spectrum values, as compared to larger microphone arrays. Second, smaller-sized microphone arrays had relatively wider main robes, reflecting a poorer spectral resolution. Enlarging the size of the microphone arrays could remediate these limitations, as is evident from the simulated results. However, it could worsen temporal detectability, as described in Figure 5. This is because the target sound could not be contained in the processing window size of the frequency analyses. The size of the processing window associated with the sampling rate of 16,000 used for the simulation contains 512 samples which is equivalent to 10.8 m. In other words, the largest microphone arrays simulated had a diameter of 20 m with a time difference equivalent to approximately 1,000 samples, which exceeded 512 samples.

**FIGURE 13:**
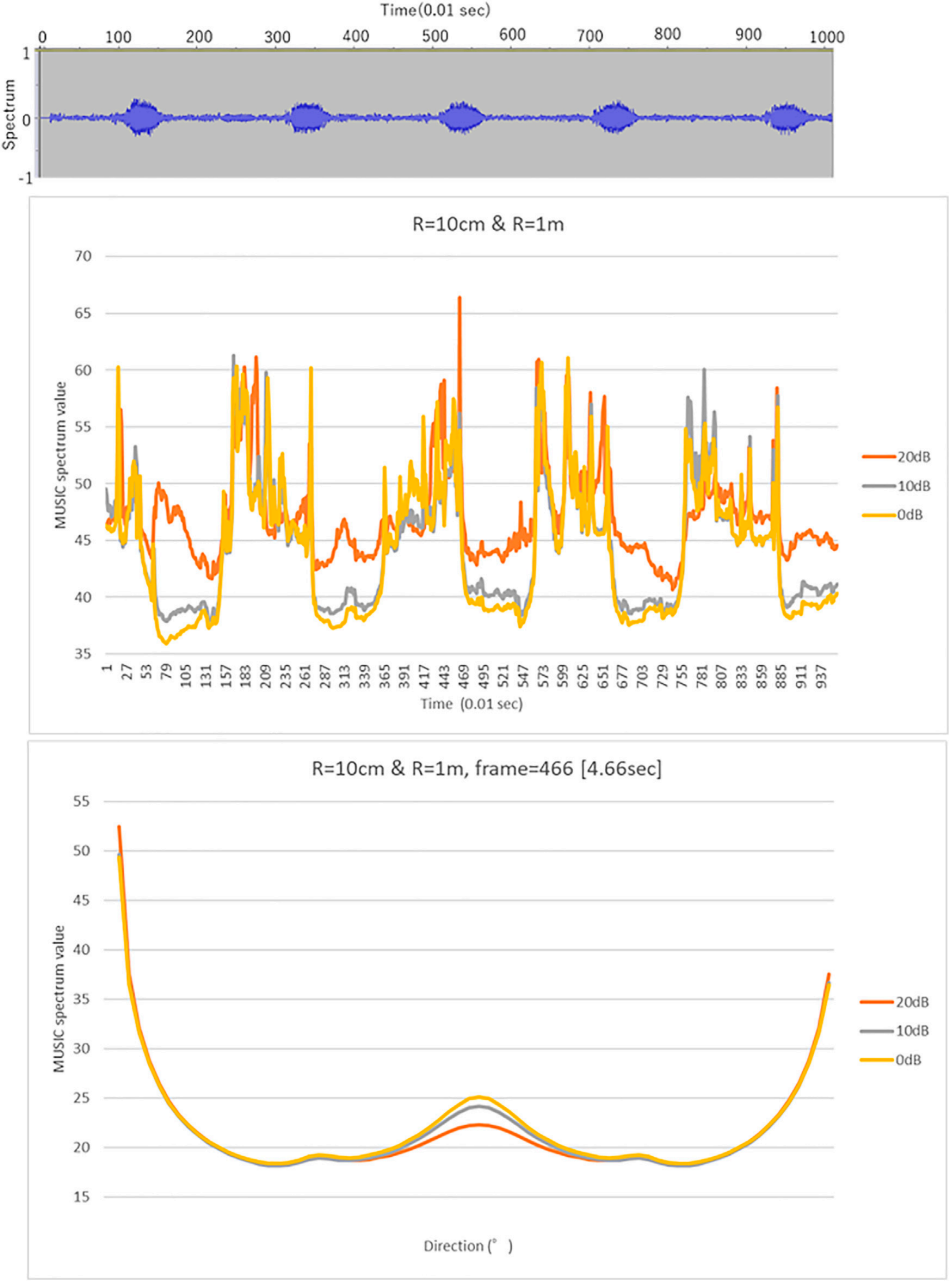
MUSIC temporal spectrum (top) and temporal spectrum (bottom) in LF for the hybrid microphone array.

One potential solution to this issue is the use of a hybrid method. Smaller microphone arrays could be used to temporally detect sound sources, and then use larger microphone arrays with finer spectral resolution to localize sounds afterwards. Figure 13 shows the localization results of a hypothetical hybrid microphone array in the LF range. This hybrid microphone array had 8 microphone nodes, four of which had a radius of 10 cm and the rest with a radius of 10 m, alternately arranged. This formation allows each microphone node to cover 90°. Second, we could lower the sampling rates to levels that matched the frequency range of the target. For example, if we lowered the sampling rate from 16,000 to 1,000 Hz, the size of the processing window for 512 samples increased from 10.8 to 160 m, thereby allowing all the sounds derived from the 8 microphone nodes of the 10 m-radius microphone array contained in this range.

Despite the technical limitations associated with microphone arrays, this study demonstrated that robot audition could successfully be applied to monitor nocturnal birds in the field. Using portable microphone arrays, we localized advertisement calls of at least two Eurasian bitterns that have an extremely low-pitched frequency range. To the authors' best knowledge, this is the first attempt to monitor the spatiotemporal distribution of vocal activity of bitterns based on localized sounds derived from a small microphone array, as compared to the ones in other similar studies, e.g., microphone array radius of 30 cm in Frommolt and Tauchert (2014) and 65–94 m in Wahlberg et al. (2003).

The results show that this technology is applicable to conduct auditory surveys of the numbers, behaviors, and microhabitat selection of nocturnal wetland birds. All the above-mentioned measurements are difficult to obtain using the other observation methods, especially in highly reverberate wetland environments. DOAs were particularly useful not only to identify two booming males, but also to distinguish actual vocalizations of each bittern from the echoes of the originals. Reverberation and echoing were most severe at a site facing the water body, surrounded by thick and tall wetland vegetation and riparian trees, resulting in false detection of the bittern. In addition to the merits of data consistency and the absence of observer bias, this observation method significantly decreases the observation cost and risk of bird monitoring in dark wetlands. The potential bittern habitat in Japan is known for its high population of grizzly bears. Future studies will include integrating DOAs from multiple microphone arrays to enable the estimation of the distance to the target bird.

Although we have shown the effectiveness of robot audition for auditory monitoring of nocturnal wetland birds that have low-frequency vocalization using small-sized microphone arrays, we should note that our recording example of Eurasian Bittern is a simple situation, relative to monitoring, and thus may not be extrapolated to other birds for three reasons. First, bitterns are widely dispersed; thus, it is easier to discriminate individuals compared to other flocking birds. Second, bitterns’ vocalizations are simply structured, loud, low, and narrow banded; thus, they travel over long distances and are relatively insensitive to deterioration during sound transmission. Third, they are less mobile when they vocalize. Most birds have more broadband vocalizations and may be in motion; thus, they are difficult to detect owing to frequency-dependent variations in the transmission loss.

The main task of this monitoring was to detect the number of territorial bitterns and their territory size. Based on the observation results and localization limit, we concluded that there were two mature males in the study area at the time of recording, both of which were booming in the southern part of the lake. There are two likely reasons why both were found only on the southern side of the lake. First, the southern shore of the lake is more isolated and contains a larger wetland area. In contrast, the northern shore is closer to residential areas and partially developed for recreational areas. The area contains fewer reed marshes. Second, the entrance to the southern part was temporarily restricted to protect the breeding of the Japanese crane. Although limited by a short observation period, higher detection rates in the southern part reflect habitat quality as well as the effective management of human interruption. Continuation of monitoring is required to further assess habitat preferences.

We have two recommendations for prioritizing the development of localization technologies. Automated species classification via machine learning is essential. Although HARKBird performs automatic sound detection and sound separation into multiple noise-reduced recordings based on the MUSIC method, we manually inspected each of the localized sounds. There are two reasons for the need for manual inspection. First, we had severe reverberation and echoes caused by the water surface surrounding the trees. Second, low-pitch sounds were similar to background environmental noises such as the rustling of leaves that occurred in a direction similar to that of the sound source. The comparison between the localized and annotated call duration revealed that the localization accuracy was highly affected by the level of surrounding noise and the distance to the microphone array. More robust methods to classify targets as well as separate targets from background noise are needed to achieve a truly automated auditory survey of bird vocalization. One potential method to improve the performance of automated sound classification is the use of beamforming in sound separation (Kojima et al., 2016). Increased performance in automated sound detection will significantly improve the usability of localization techniques for auditory monitoring of birds in the real field with many environmental noises. A more robust classification method in noisy surroundings will not only help delineate target sounds from other coexisting species, but also capture the soundscape.

Second, there is a need for recording equipment that is truly water-proofed, portable, and self-synchronizing. Many of the fields, such as the wetland environment in this study, are wet and suffer from moisture condensation. Although our recording gear, TAMAGO, is simple and water-proofed, the USB cables connecting TAMAGO, Raspberry-pi, and the mobile battery are partially exposed. This makes it not optimal for use in wetlands that are highly misty. Paradoxically, many rare species that biologists need to survey are found in misty environments. The degree of waterproofing coincides with the size and weight of the equipment, yet carrying a large and heavy equipment is not realistic in the poor footing or remote fields. Self-synchronization is another challenge. In our study, we synchronized Raspberry Pi by tethering it to the cell phone, yet delays occurred randomly. GPS synchronization could be a solution, but the current cost of the device will limit its availability.

Ultimately, automated sound localization on mobile robots has great potential for collecting auditory data at larger and longer spatiotemporal scales, with finer resolution and higher accuracy compared to human observers. As demonstrated in our study, it can detect the numbers, movements of individuals, and habitat use of the target. This method is particularly useful for monitoring rare species in an unintrusive manner, while reducing the observation cost and risks to the human observer.

## Data Availability

The original contributions presented in the study are included in the article/Supplementary Material, further inquiries can be directed to the corresponding author.
